# Pak2 reduction induces a failure of early embryonic development in mice

**DOI:** 10.1186/s12958-021-00865-3

**Published:** 2021-12-09

**Authors:** Juan Zeng, Nengqing Liu, Yinghong Yang, Yi Cheng, Yuanshuai Li, Xiaoxia Guo, Qian Luo, Lifen Zhu, Hongmei Guan, Bing Song, Xiaofang Sun

**Affiliations:** 1 Department of Obstetrics and Gynecology, Key Laboratory for Major Obstetric Diseases of Guangdong Province, The Third Affiliated Hospital of Guangzhou Medical University, Guangzhou, Guangdong China; 2 Key Laboratory of Reproduction and Genetics of Guangdong Higher Education Institutes, Guangzhou, Guangdong China

**Keywords:** Apoptosis, Embryo, Oxidative stress, Spindle assembly, Pak2

## Abstract

**Background:**

The quality of the early embryo is vital to embryonic development and implantation. As a highly conserved serine/threonine kinase, p21-activated kinase 2 (Pak2) participates in diverse biologic processes, especially in cytoskeleton remodeling and cell apoptosis. In mice, Pak2 knock out and endothelial depletion of Pak2 showed embryonic lethality. However, the role of Pak2 in preimplantation embryos remains unelucidated.

**Methods:**

In the present work, Pak2 was reduced using a specific small interfering RNA in early mouse embryos, validating the unique roles of Pak2 in spindle assembly and DNA repair during mice early embryonic development. We also employed immunoblotting, immunostaining, in vitro fertilization (IVF) and image quantification analyses to test the Pak2 knockdown on the embryonic development progression, spindle assembly, chromosome alignment, oxidative stress, DNA lesions and blastocyst cell apoptosis. Areas in chromatin with γH2AX were detected by immunofluorescence microscopy and serve as a biomarker of DNA damages.

**Results:**

We found that Pak2 knockdown significantly reduced blastocyst formation of early embryos. In addition, Pak2 reduction led to dramatically increased abnormal spindle assembly and chromosomal aberrations in the embryos. We noted the overproduction of reactive oxygen species (ROS) with Pak2 knockdown in embryos. In response to DNA double strand breaks (DSBs), the histone protein H2AX is specifically phosphorylated at serine139 to generate γH2AX, which is used to quantitative DSBs. In this research, Pak2 knockdown also resulted in the accumulation of phosphorylated γH2AX, indicative of increased embryonic DNA damage. Commensurate with this, a significantly augmented rate of blastocyst cell apoptosis was detected in Pak2-KD embryos compared to their controls.

**Conclusions:**

Collectively, our data suggest that Pak2 may serve as an important regulator of spindle assembly and DNA repair, and thus participate in the development of early mouse embryos.

## Background

The early embryonic development of mammals is activated when a mature oocyte (*MII*) is fertilized by a mature spermatozoon [1]. After fertilization, the zygote undergoes cleavage divisions from the 2-cell to blastocyst stages, at which point the embryos are implanted into the mother’s uterus on embryonic day 4.5 in mice [2–4]. We now appreciate that embryonic development depends upon precise spatiotemporal regulation of gene expression [5].

Paks (p21-activated kinase) comprise an evolutionarily conserved group of serine/threonine kinases that regulate diverse cellular activities [6]. The mammalian Pak family consists of six members and is divided into two groups: group I is composed of Pak1, Pak2, and Pak3—with Pak1 and Pak3 being tissue-specific and showing the highest levels in brain—whereas Pak2 is ubiquitous [7]; group II is composed of Pak4, Pak5, and Pak6 [8]. The most fundamental and vital function of Pak2 is to regulate the remodeling of the cytoskeleton [9]. Pak2 can be activated by Cdc42 (GTP) and under various stress conditions, and cleaved caspase 3 also constitutively activates Pak2 during the apoptotic process [10]. Under low-amplitude physiologic forces, Pak2 is protected from proteolysis so as to ensure cellular survival, but under higher-amplitude forces Pak2 is left unprotected and stimulates apoptosis [11]. In addition, Pak2 has been reported to be an important regulator of cellular senescence and organismal aging [12]. Moreover, Pak2 acts as a molecular switch for cytostasis and apoptosis in response to different types and levels of stress, with broad physiologic and pathologic relevance [10]. Pak2 cardiac-deleted mice (Pak2-CKO) manifested endoplasmic reticulum stress, cardiac dysfunction, and severe cell death [13]; and Pak2 knockout mice showed embryonic lethality on embryonic day 8.5 (E8.5) due to multiple developmental abnormalities [7, 14, 15].

Pak2 affects wide range of biological processes and Pak2-null mice are embryonic lethality [15, 16]. However, the role for Pak2 in early mouse embryonic development remains unclear. In the current study, we explored Pak2 function during the development of early mouse embryos by using a small interfering RNA (siRNA) to silence the Pak2 gene. Our findings indicate that Pak2 is involved in the control of developmental progression and potential of the early embryos of mouse.

## Materials and methods

### Mice

We used ICR mice, 6–8 weeks of age, in the present study. Experiments were approved by the Third Affiliated Hospital of Guangzhou Medical University Animal Care and Use Committee and conducted in accordance with the guiding principles of the institution.

### Antibodies and chemicals

All the chemicals and reagents were purchased from Sigma unless stated otherwise. Rabbit polyclonal anti-Pak2 antibody (Cat#: ab76293) and anti-γ-H2AX (phosphor S139) antibody (Cat#: ab81299) were obtained from Abcam (Cambridge, MA, USA); mouse monoclonal anti-α-tubulin-FITC antibody (Cat#: F2168) from Sigma (St. Louis, MO, USA); Alexa Fluor 488 goat anti-rabbit IgG (H + L) (Cat#: A11008) and CM-H2DCFDA (Cat#: C6827) from Thermo Fisher Scientific Life Technologies (Massachusetts, MA, USA); an In Situ Cell Death Detection Kit (Cat#:11684817910) was purchased from Roche (Basel, Switzerland); and HRP-conjugated Affinipure Goat Anti-Rabbit IgG (H + L) (Cat#:SA00001–2), HRP-conjugated Affinipure Goat Anti-Mouse IgG (H + L) (Cat#:SA00001–1), and GAPDH Monoclonal Antibody (Cat#:60004-1 g) were purchased from Proteintech (Wuhan, China).

### In vitro fertilization and embryo culture

In vitro fertilization (IVF) was performed according to methods described previously [17]. Adult mice (> 8 weeks of age) were used for in vitro fertilization (IVF). Human tubal fluid fertilization medium (HTF; Santa Ana, CA, USA; Cat#: 90125) containing 1% bovine serum albumin (BSA; Sigma, St. Louis, MO, USA; Cat#: A1933-25G) was used as IVF culture medium. Briefly, spermatozoa were collected from the epididymides of adult males and incubated in droplets of IVF culture medium for 1 h at 37 °C under 5% CO2 in humidified air for capacitate. Female mice were injected with 7.5 IU of equine chorionic gonadotropin (eCG; Teikoku Zoki, Tokyo, Japan), and 48 h later they were injected with 7.5 IU of human chorionic gonadotropin (hCG; Teikoku Zoki, Tokyo, Japan). Thirteen hours after hCG injection, the cumulus-oocyte complexes (COC) were isolated from the oviduct, and capacitated spermatozoa were placed into HTF-droplets containing COCs for co-incubation of at least 5 h. Fertilized zygotes were washed with potassium simplex optimization medium (KSOM; Sigma, St. Louis, USA; Cat# MR-106-D) and incubated in KSOM medium under paraffin oil. Embryos at 8 h, 24 h, 48 h, 56 h, 96 h after IVF were collected as at pronucleus, 2-cell, 4-cell, 8-cell and blastocysts stage, respectively.

### siRNA knockdown

To explore the functions of Pak2 in early mouse embryos, specific Pak2-siRNA and negative control siRNAs were obtained from Shanghai GenePharma Co, Ltd. The siRNAs were diluted to 1 mM with RNase-free ddH2O and stored in a − 80 °C refrigerator. When microinjection, the siRNAs were diluted to 20 μM and approximately 5–10 pL Pak2-siRNA solution was microinjected into the zygote. Microinjections of small interfering RNAs (siRNA) with a Narishige microinjector were used to knockdown Pak2 in zygotes. The Pak2-siRNA pairs that we used were as follows: forward strand, siRNA#1, 5′-CCGUGUGCAGAGAGUGUUUTT- 3′; reverse-strand, 5′-AAACACUCUCUGCACACGGTT- 3′; siRNA#2, 5′-AAUCACAGUUUGAAACCUUTT- 3′; reverse-strand, 5′-AAGGUUUCAAACUGUGAUUGG- 3′). A nonspecific siRNA was used as a negative control: forward strand, 5′-UUCUCCGAACGUGUCACGUTT- 3′; reverse-strand, 5′-ACGUGACACGUUCGGAGAATT- 3′).

### Western immunoblotting analysis

A total of 70 two-cell embryos were lysed in 12 μL of Laemmli sample buffer (95 μL of loading buffer contained 5 μL of β-mercaptoethanol) and denatured at 100 °C for 5 min. Protein samples (70 embryos each sample per lane) were separated on 12% SDS-PAGE gels and transferred to PVDF membranes, blocked in 5% skim milk diluted with PBS-Tween 20 (0.1%, vol/vol) for 1 h, and then incubated with primary antibody overnight at 4 °C (Pak2, 1:1000; GAPDH, 1:2000). After at least three washes with PBS-Tween 20, membranes were incubated with secondary antibody for 1 h at room temperature. After an additional three washes with PBS-Tween 20, the protein bands were detected with ECL Plus Western Blotting Detection System (GE Healthcare, Piscataway, NJ, USA).

### Immunofluorescence

Early mouse embryos were fixed in 4% paraformaldehyde (Sigma, St. Louis, USA; Cat# 158127-100G) for 30 min, permeabilized with 0.1% Triton X-100 (Sigma, St. Louis, USA; Cat# T8787-100ML) for 20 min, and then blocked in PBS-BSA (1%, wt/vol) for 60 min. Embryos were incubated with the primary antibodies anti-Pak2 (1:200) and anti-γH2AX (1:300), and anti-α-tubulin FITC-labeled antibodies (1:200) overnight at 4 °C. After three washes with PBS-BSA, samples were incubated with secondary antibodies (1:100) for 1 h at room temperature, and chromosomes were co-stained with propidium iodide (PI; Sigma, St. Louis, USA; Cat# 81845-25MG) or Hoechst 33342 (Sigma, St. Louis, USA; Cat# B2261-25MG) for 10 min. Finally, samples were mounted on glass slides with antifade medium (Vectashield, Burlingame, CA; Cat# H-1000) and then examined via laser-scanning confocal microscopy (LSCM, Leica SP8, Germany).

### Measurement of ROS levels

Changes in intracellular ROS content were determined using CMH2DCFDA (Life Technologies, Invitrogen TM, Carlsbad, CA, USA; Cat#: C6827). Two-cell embryos were incubated for 20 min at 37 °C in M2 medium (Sigma, St. Louis, USA; Cat# M7167) containing 5 mM CMH2DCFDA, and after three washes in KSOM medium, embryos were placed on a confocal dish with a microdrop of M2 medium. Images of embryonic fluorescence emission were captured under LSCM and analyzed using ImageJ software.

### Statistical analysis

All experiments were repeated at least three times. Results are presented as means ± one standard deviation and analyzed by Student’s *t* test. We employed SPSS20.0 for statistical analyses, and *P* < 0.05 was considered to be statistically significant.

## Results

### Subcellular localization of Pak2 in early embryos

**Fig. 1:**
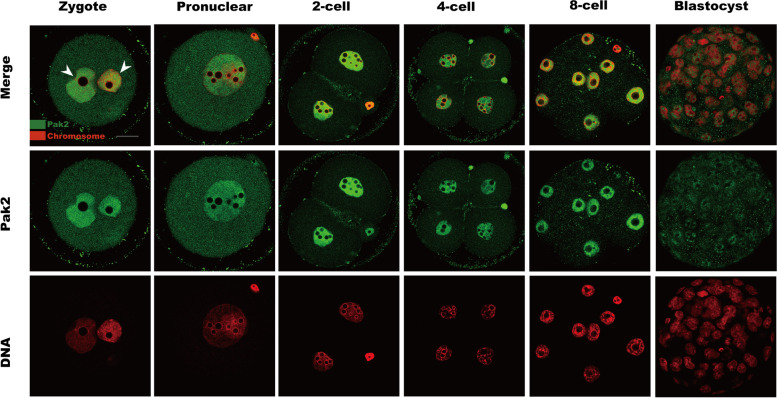
Subcellular localization of Pak2 in early mouse embryos. Zygotes; pronuclear-stage, 2-cell, 4-cell, and 8-cell embryos; and blastocysts were labeled with a Pak2 antibody (green) and counterstained with propidium iodide (PI; red) to visualize DNA. Arrowheads indicate the accumulated Pak2 signal. Scale bars, 20 μm; Pak2, p21-activated kinase 2

The subcellular localization of Pak2 in zygotes and pronuclear-, two-cell-, four-cell-, eight-cell-, and blastocyst-stage embryos was investigated by immunofluorescence staining. Our results revealed that Pak2 signals were distributed throughout the entire embryo, with strong accumulation in the nucleus relative to the cytoplasm (Fig. 1). This particular pattern of Pak2 protein localization implied that it may function in early embryonic development.

### Pak2 reduction compromises early developmental potential of embryos

**Fig. 2:**
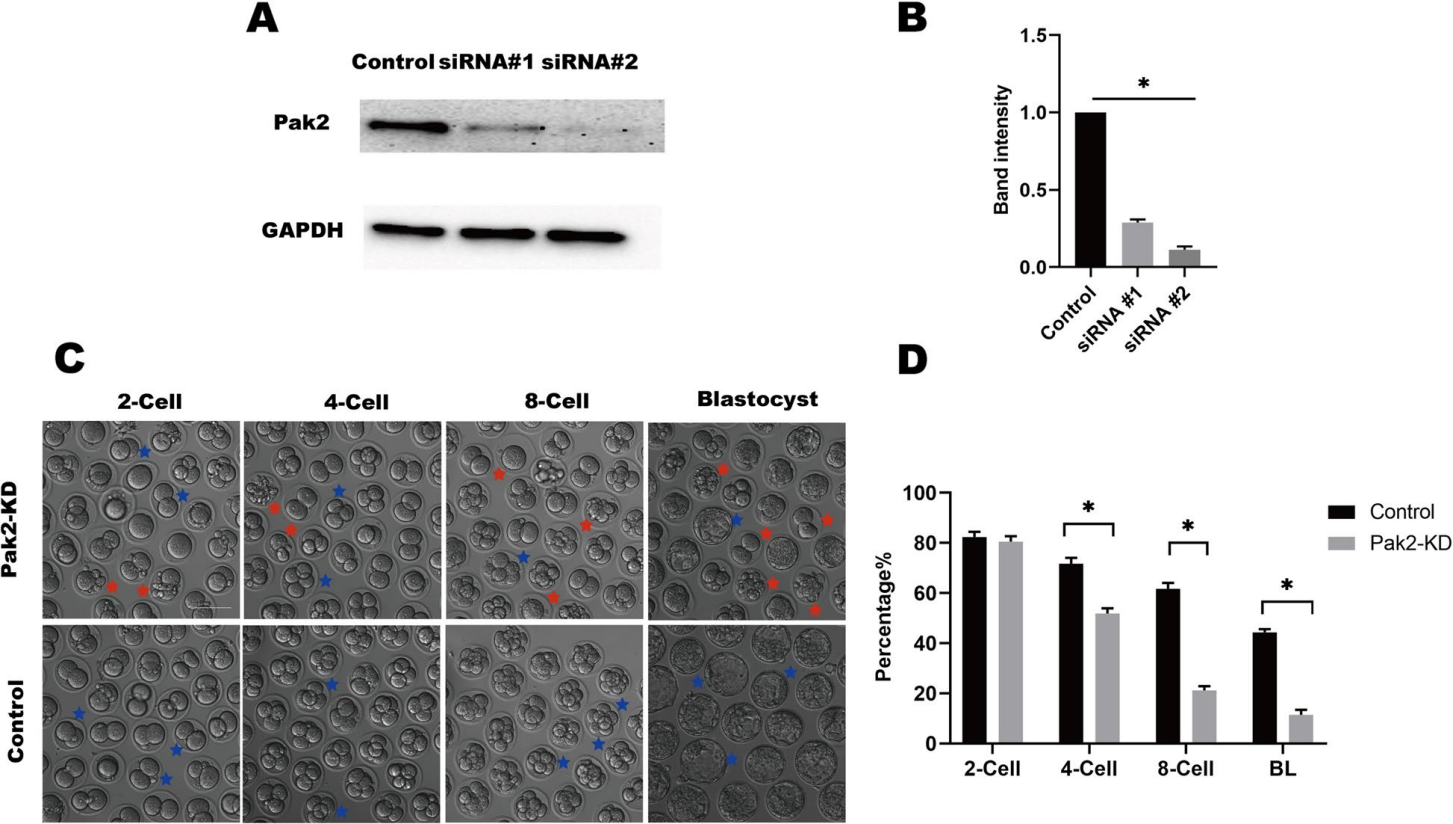
Pak2 knockdown disrupts the developmental potential of early embryos. In vitro culture of zygotes microinjected with Pak2-siRNA and analysis of developmental progression. **A**, With GAPDH as a loading control, the efficiency of Pak2 knockdown was confirmed by western blot analysis. **B**, Band intensity was calculated using ImageJ software (NIH, USA), and the ratio of Pak2/GAPDH expression was normalized and the values are indicated. **C**, Representative bright-field images of 2-cell embryos, 4-cell embryos, 8-cell embryos, and blastocysts from control and Pak2-KD groups. Bule asterisks indicate examples of normal morphology; red asterisks indicate examples of abnormal morphology (scale bars, 100 μm). **D**, Quantification analysis of the rate of 2-cell, 4-cell, 8-cell and blastocyst in control embryos and Pak2-KD embryos. (2-cell: 82.3 ± 1.76%, *n* = 118 vs. 80.5 ± 1.82%, *n* = 127, respectively; 4-cell: 71.7 ± 1.85%, *n* = 103 vs. 51.8 ± 1.73%, *n* = 82, respectively; 8-cell: 61.7 ± 1.93%, *n* = 89 vs. 21.2 ± 1.36%, *n* = 33, respectively; blastocyst: 44.3 ± 1.13%, *n* = 63 vs. 11.47 ± 1.62%, *n* = 18, respectively). The graph shows the mean perentage ± SD of the results obtained in three independent experiments. *Significantly different (*p* < 0.05). Pak2, p21-activated kinase 2; SD, standard deviation; Pak2-KD, Pak2 knockdown; BL, blastocyst

We next aimed to illustrate the role of Pak2 in early embryonic development of mice. We microinjected specifically designed siRNAs into zygotes to investigate the function of Pak2 during embryonic development, and demonstrated that endogenous Pak2 proteins were reduced by approximately 80% or 90% as verified by western blotting analysis (Fig. 2A and B), since the siRNA#2 knockdown is more efficient, we chose it for subsequent experiments. There were no obvious differences in the morphology of the early mouse embryos between control and Pak2- KD groups (Fig. 2C). However, Pak2-reduction in embryos showed significant developmental delays or cytoplasmic fragmentation (red asterisk) in 4-cell-, 8-cell-, and blastocyst-stage embryos,

Pak2-knockdown also markedly reduced the rate of blastocyst formation (2-cell: 82.3 ± 1.76%, *n* = 118, control vs. 80.5 ± 1.82%, *n* = 127, Pak2-KD, *p* > 0.05; 4-cell: 71.7 ± 1.85%, *n* = 103, control vs. 51.8 ± 1.73%, *n* = 82, Pak2-KD, *p* < 0.05; 8-cell: 61.7 ± 1.93%, *n* = 89, control vs. 21.2 ± 1.36%, *n* = 33, Pak2-KD, *p* < 0.05; blastocyst: 44.3 ± 1.13%, *n* = 63, control vs. 11.47 ± 1.62%, *n* = 18, Pak2-KD, *p* < 0.05; Fig. 2C and D). The above observations suggested that the developmental potential of Pak2-KD embryos was impaired during in vitro culture.

### Attenuated Pak2 adversely affects spindle assembly and chromosomal congression in mouse embryos

**Fig. 3:**
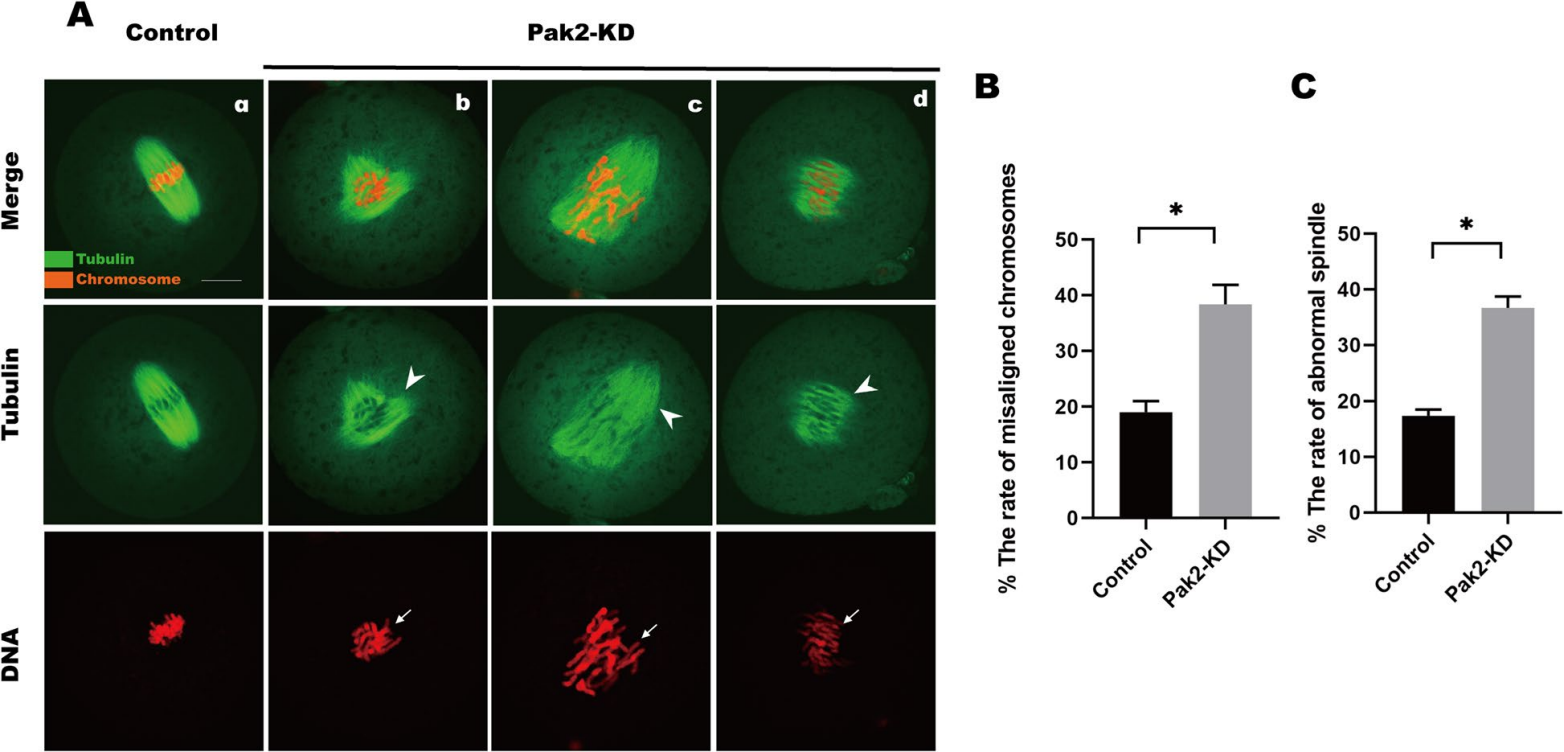
Pak2 knockdown affects spindle morphology and chromosomal alignment in early mouse embryos. (**A**) Early mouse embryos at metaphase were stained with anti-α-tubulin (green) and counterstained with propidium iodide (PI) to visualize chromosomes (red). In contrast to the control group, embryos in the Pak2-KD group showed a variety of defects, including multipolar and nonpolar spindles. Arrows indicate spindle defects and arrowheads indicate chromosome misalignment in zygotes. The chromosomes in embryos of the Pak2-KD group were severely misaligned (bar = 20 μm). (**B**) A significantly higher proportion of embryos in the Pak2-KD group exhibited spindle defects relative to the control group (37.0 ± 3.89%, *n* = 64 vs. 17.9 ± 1.24%, *n* = 56; respectively). (**C**) The incidence of chromosomal misalignment was also higher in embryos in the Pak2-KD treatment group compared to the control group (38.8 ± 11.77%, *n* = 67 vs. 19.5 ± 4.62%, *n* = 73; respectively). Data are expressed as the mean ± SD from three independent experiments. *Significantly different (*p* < 0.05). Pak2, p21-activated kinase 2; SD, standard deviation; Pak2-KD, Pak2 knockdown

Since Pak2 has been implicated in regulating cytoskeleton dynamics [18], we herein explored the role of Pak2 in mitosis by treating early embryos with small interfering RNAs, and immunostaining them with an anti-α-tubulin antibody to show spindle morphology and counterstaining with PI to observe chromosomes. Most embryos in the control group showed complete bipolar spindles and well-aligned chromosomes (Figure 3Aa). However, the spindles of embryos in the Pak2-KD group revealed multiple defects, such as multipolar (Figure 3Ab, arrows), non-polar spindles (Figure 3Ad, arrows). Moreover, the majority of embryos in the Pak2-KD group exhibited severe chromosomal aberrations (Figure 3Abc, arrowheads). The incidence of embryonic spindle defects in the Pak2-KD group was significantly higher than that in the control group (37.0 ± 3.89%, *n* = 64, Pak2-KD vs. 17.9 ± 1.24%, *n* = 56, control, *p* < 0.05; Fig. 3B), as was the incidence of chromosomal aberrations (38.8 ± 11.77%, *n* = 67, Pak2-KD vs. 19.5 ± 4.62%, *n* = 73, control, *p* < 0.05; Fig. 3C).

### Reduced Pak2 induces elevated ROS levels in mouse embryos

**Fig. 4:**
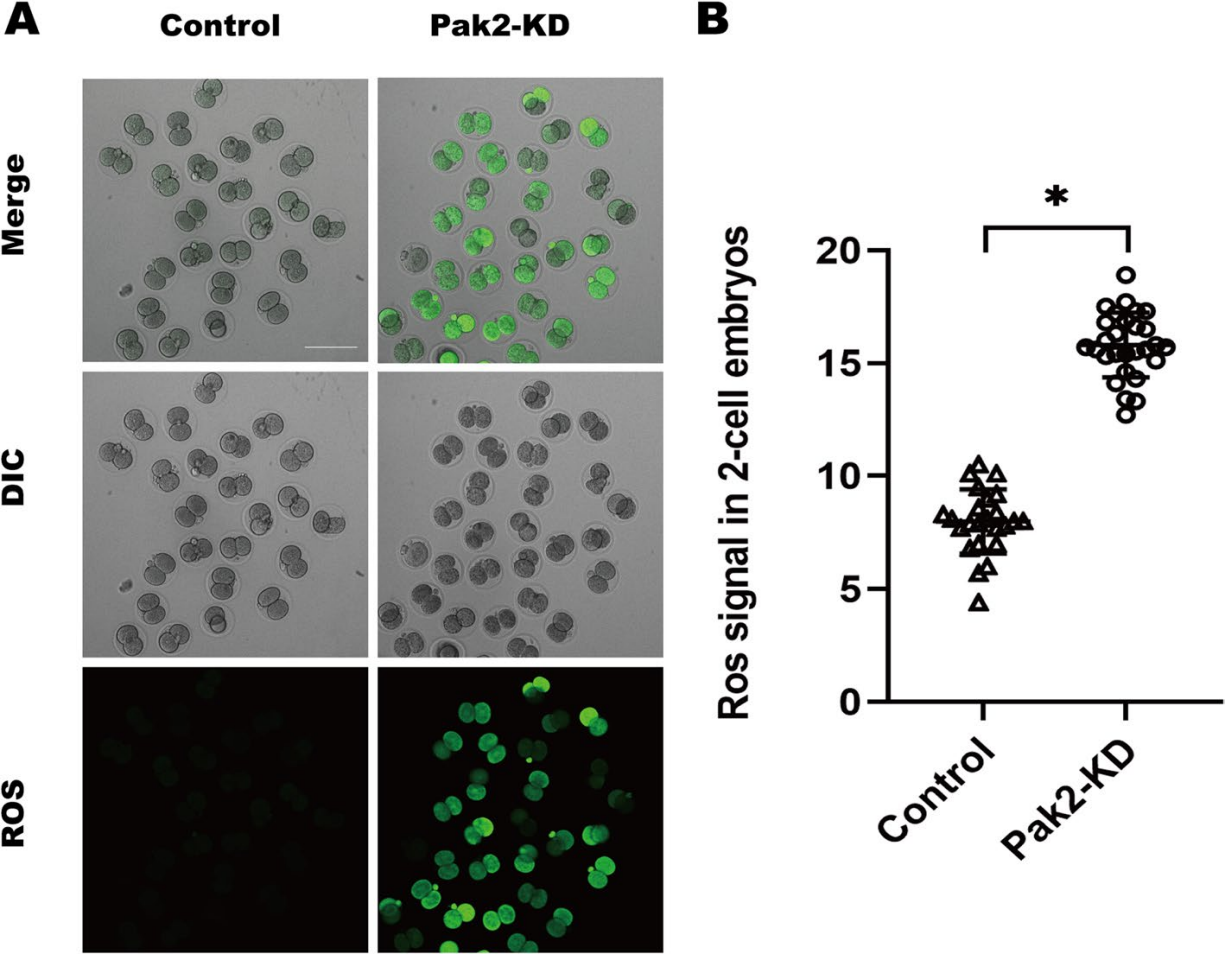
Pak2 knockdown elevates ROS levels in embryos. (**A**) Representative images of CM-H2DCFDA fluorescence (green) in 2-cell embryos from control and Pak2-KD groups (*n* = 24 for control group and *n* = 28 for Pak2-KD group; scale bars, 120 μm). (**B**) Quantitative analysis of fluorescence intensity in control and Pak2-KD embryos (7.9 ± 1.39, n = 24 vs. 15.8 ± 1.41, *n* = 28, respectively). Data are expressed as the mean ± SD from three independent experiments. *Significantly different (*p* < 0.05). Pak2, p21-activated kinase 2; Pak2-KD, Pak2 knockdown; SD, standard deviation; ROS, reactive oxygen species

It was demonstrated that Pak2 inhibition induced reactive oxygen species overproduction and mitochondrial-JNK pathway activation [19]. As production of ROS is a major measure of mitochondrial function [20], we therefore asked whether Pak2 knockdown influences mitochondrial status in the early embryos of mice. To address this question, 2-cell embryos were collected from control and Pak2-KD groups and stained with CM-H2DCFDA fluorescent dye for the assessment of ROS generation. Compared with the control group, Pak2-KD treatment significantly increased the levels of ROS in 2-cell embryos (Fig. 4A) as determined by mean fluorescence intensity (7.9 ± 1.39, *n* = 24, control vs. 15.8 ± 1.41, *n* = 28, Pak2-KD, *p* < 0.05; Fig. 4B). These findings imply that Pak2 participates in the regulation of redox homeostasis in mouse preimplantation embryos.

### Decreased Pak2 causes the DNA damage in early embryos

**Fig. 5:**
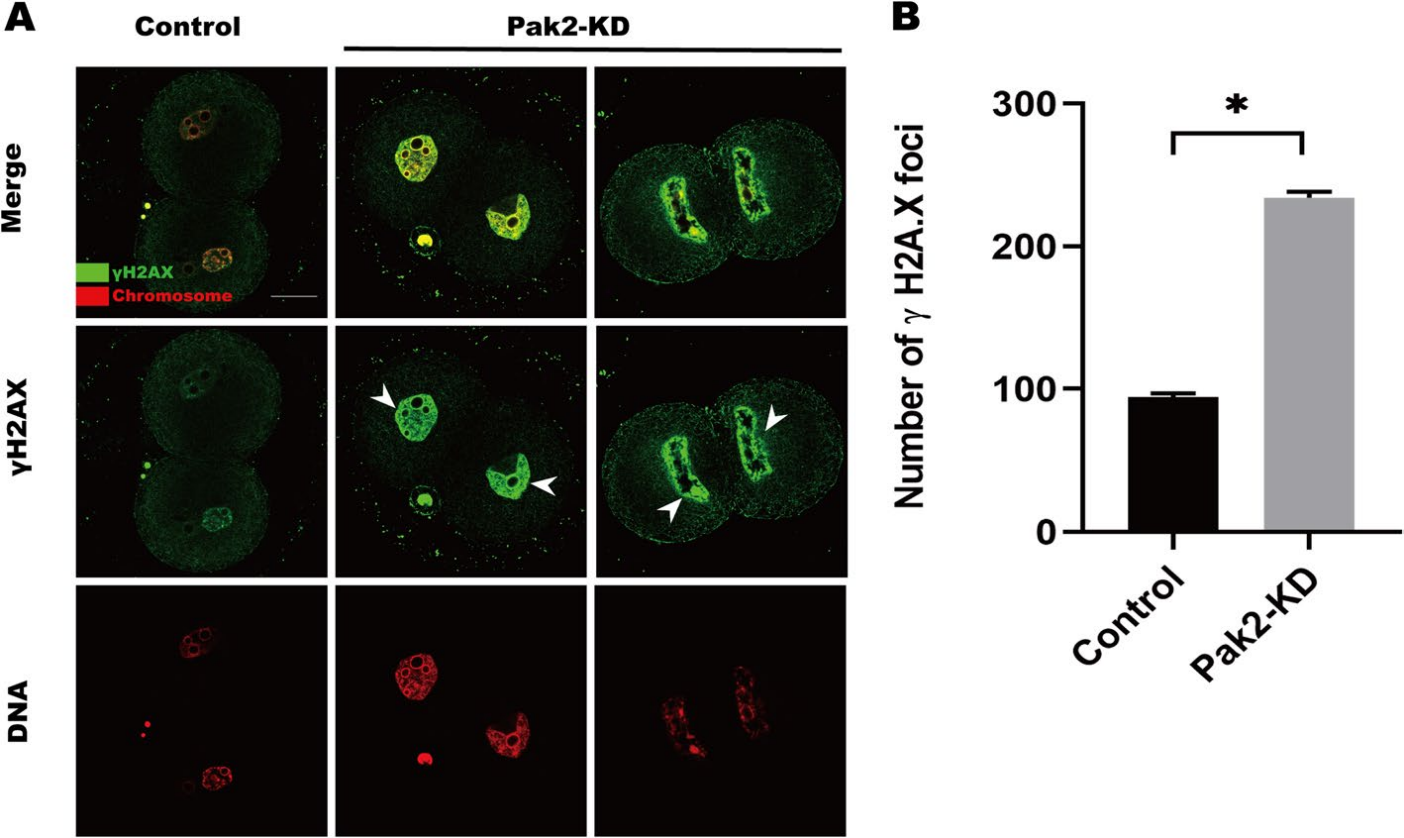
Pak2 knockdown results in the accumulation of γH2AX during preimplantation embryonic development. (**A**) Two-cell embryos were immunostained with an anti-γH2AX antibody to detect DNA damage (green) and counterstained with PI for DNA (red). Arrows indicate the increased DNA damage in embryos (scale bars, 20 μm). (**B**) Quantification of the numbers of γH2AX foci in control and Pak2-KD group embryos (94.3 ± 2.59, *n* = 32 vs. 234.0 ± 4.31, *n* = 37; respectively). Data are expressed as the mean ± SD from three independent experiments. *Significantly different (*p* < 0.05). Pak2, p21-activated kinase 2; Pak2-KD, Pak2 knockdown; SD, standard deviation; PI, propidium iodide

Histone H2AX phosphorylation (γH2AX) can be triggered by DNA double-strand breaks (DSBs) [21]. When cellular DSBs occur, H2AX is rapidly phosphorylated in the damaged chromatin, and this activity is localized in nuclear foci [22]. In the present study, γ-H2AX-recognizing antibodies were used to quantify DSBs, and we found that γH2AX foci in Pak-KD embryos (Fig. 5A, arrows) were significantly increased compared to control embryos (94.3 ± 2.59, *n* = 32, control vs. 234.0 ± 4.31, *n* = 37, Pak2-KD, *p* < 0.05; Fig. 5A and B). These results imply that Pak2 is essential for genomic integrity of the early embryo.

### Pak2 knockdown enhances apoptosis of blastocyst in mouse embryos

**Fig. 6:**
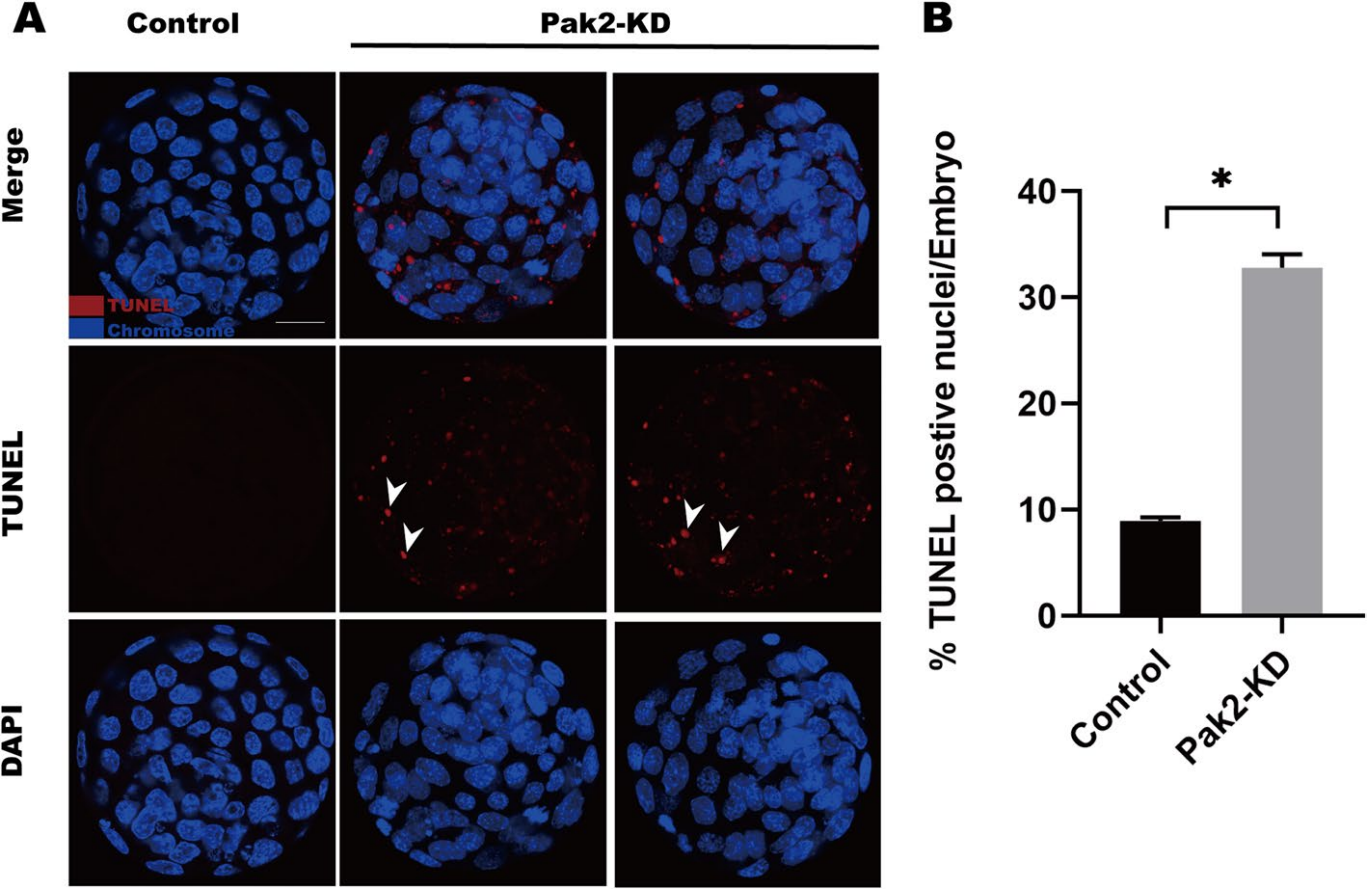
Pak2-KD induces apoptosis of early mouse embryos. (**A**) TUNEL analysis of control and Pak2-KD embryos. Embryos were labeled with Hoechst 33342 (blue) for DNA and TUNEL used to assess fragmented DNA (red). Arrows point to apoptotic cells in blastocysts (scale bars, 20 μm). (**B**) Quantification of control and Pak2-KD group blastocysts with TUNEL- positive nuclei (8.9 ± 0.32, *n* = 50 vs. 32.8 ± 1.25, *n* = 37; respectively). Data are expressed as mean percentage ± SD from three independent experiments. *Significantly different (*p* < 0.05). Pak2, p21-activated kinase 2; SD, standard deviation; Pak2-KD, Pak2 knockdown; TUNEL, terminal deoxynucleotidyl transferase dUTP nick-end labeling

Considering the elevated DNA damage in Pak2-KD embryos, we conducted TUNEL assays to evaluate the apoptotic status in blastocyst-stage embryos. As shown in Fig. 6A, TUNEL-positive nuclei were almost undetectable in control embryos, but we readily observed TUNEL-positive cells in PAK2-KD blastocysts (Fig. 6A, arrows). Quantitative analysis further revealed a significantly increased percentage of Pak2-KD embryos with TUNEL-positive nuclei relative to controls (8.9 ± 0.32, *n* = 50, control vs. 32.8 ± 1.25, *n* = 37, Pak2-KD, *p* < 0.05; Fig. 6B).

## Discussion

Pak2, as a highly conserved serine/threonine protein kinase, plays a significant role in cell motility, survival, mitosis, and apoptosis [23]. In view of the subcellular localization pattern in early embryos of mice (Fig. 1), we speculated on its involvement in chromatin-related cellular events. To validate our hypothesis, early mouse embryos treated with a Pak2-specific siRNA exhibited a significant increase in abnormal spindle assembly and chromosomal aberrations that contributed to their abnormal early development (Fig. 3). Pak2 has been reported to regulate cytoskeletal dynamics in diverse cell types [9, 18, 24, 25]. In a recent study, investigators noted that inactivation of Pak2 caused oxidative stress [26], and that Pak2 was highly activated when mammalian cells were treated with hydrogen peroxide [10]. In glioblastoma A172 cell, Pak2 inhibition induced reactive oxygen species overproduction, mitochondria-JNK pathway activation [19]. In our study, ROS levels were dramatically increased in early embryos when Pak2 activity diminished (Fig. 4), which suggested mitochondrial dysfunction. ROS exert detrimental effects on DNA, RNA, proteins, lipids, and other cellular components — consequently disturbing multiple biologic events that include cellular metabolism, apoptosis, and senescence [27]. Excessive ROS is thus detrimental to normal embryonic development [28].

It was reported that ROS comprised an important factor causing intracellular DNA lesions [29]. γH2AX has been diffusely serve as a biomarker when detecting DNA damage in preimplantation embryos [30]. γH2AX accumulation in high-fat diet (HFD) mice zygotes and SETD2-KD mice embryos, the abnormal accumulation of γH2AX in early embryos contributed to defects in embryonic development, however, a small percentage of embryos still developed to the blastocyst stage [31, 32]. Shortage of conventional G1/S and G2/M checkpoints in the mice zygote mean that embryos carrying extensive DNA lesions can still progress through development, before the establishment of a functional apoptotic pathway in the latter stages of preimplantation embryonic development [33, 34]. In the present study, the accumulation of phosphorylated γH2AX was observed in the Pak2-KD embryos (Fig. 5), which indicated increased DNA damage; and continuous DNA lesions compromise the integrity of the genome [35], with genome stability critical for the survival, growth, and normal functioning of organisms [36–38]. ROS induce DNA-base damage, and single- and double-stranded breaks (DSBs) [39]; with DSBs constituting the most dangerous type of DNA lesion in cells [40–43]. To maintain stability, then, the delay or arrest of the cell cycle must occur to allow sufficient time for effective DNA repair [44]. For example, Pak2 dysfunction-induced cell-cycle arrest at the G1 phase caused p27Kip1 accumulation [45]. Our results also revealed that Pak2 reduction resulted in delayed embryonic development and reduced blastocyst-formation rate (Fig. 2).

When the extent of DNA lesions exceed the capacity for recovery, mitosis does not occur and cells undergo apoptosis, senescence, or death [22]. Pak2 is a kinase that can be cleaved by caspase 3 during apoptosis and occupies a dual role in apoptosis: full-length Pak2 then inhibits pro-apoptotic events by phosphorylating Bad28, whereas proteolytic activation of Pak2 p34 leads to apoptosis [11, 45]. When apoptosis was induced in MCF-7 cells with tumor necrosis factor-a (TNF-a) or Jurkat cells with C2 ceramide, Pak2 cleavage was also observed [46]. In adult endothelial cells, Pak2 loss leads to severe apoptosis and acute angiogenic defects, and the absence of Pak2 in the endothelium leads to early embryonic lethality due to flawed blood vessel formation [7]. Cytoplasmic fragmentation of early embryos is one of the hallmarks of apoptosis, it was first observed at mice 2-cell embryos [33, 47]. Our results also showed that the Pak2-KD embryos had a significant cytoplasmic fragmentation (Fig. 2C).

Pak2 plays a vital role in maintenance of endothelial barrier, endothelial cell (EC) migration and angiogenesis [7, 48]. In mice, homozygous Pak2 KO causes embryonic lethality at E8.5 and endothelial depletion of Pak2 leads to early embryo lethality at E9.5, both embryo death events were associated with angiogenesis defects [7, 15]. In adult endothelial cells, Pak2 loss leads to severe apoptosis and acute angiogenic defects, and the absence of Pak2 in the endothelium leads to early embryonic lethality due to flawed blood vessel formation, angiogenesis defects may be the result of the severe apoptotic events induced by Pak2 deletion [7]. We noted an increased number of apoptotic blastocysts in the Pak2-KD group in the current study (Fig. 6).

Collectively, these data signified that reduction of Pak2 leads to aberrations of embryonic development due to defects in chromosome congression, spindle assembly, increased levels of Reactive Oxygen Species (ROS), DNA lesions and apoptosis.

## Conclusions

In summary, our data indicated that Pak2, as a regulator of spindle assembly, DNA repair and apoptosis, plays a role in the developmental competence of mouse preimplantation embryos.

## Data Availability

All data generated or analyzed during this study are available from the corresponding author on reasonable request.

## Abbreviations

Pak2: P21-activated kinase 2
Pak2-KD: Pak2 knockdown
ROS: Reactive oxygen species
PI: Propidium iodide
TUNEL: Terminal deoxynucleotidyl transferase dUTP nick-end labeling
BL: Blastocyst
